# Comprehensive evolutionary analysis of the *Anthroherpon* radiation (Coleoptera, Leiodidae, Leptodirini)

**DOI:** 10.1371/journal.pone.0198367

**Published:** 2018-06-08

**Authors:** Iva Njunjić, Adrien Perrard, Kasper Hendriks, Menno Schilthuizen, Michel Perreau, Vincent Merckx, Michel Baylac, Louis Deharveng

**Affiliations:** 1 Institut de Systématique, Évolution et Biodiversité, ISYEB – UMR 7205 CNRS, MNHN, UPMC, EPHE, Muséum national d’Histoire naturelle, Sorbonne Universités, Paris, France; 2 Naturalis Biodiversity Center, Leiden, The Netherlands; 3 Department of Biology and Ecology, Faculty of Sciences, University of Novi Sad, Novi Sad, Serbia; 4 Groningen Institute for Evolutionary Life Sciences, University of Groningen, Groningen, The Netherlands; 5 Institute for Biology Leiden, Leiden University, Leiden, the Netherlands; 6 IUT Paris Diderot, Université Paris Diderot, Sorbonne Paris Cité, Paris, France; Sichuan University, CHINA

## Abstract

The genus *Anthroherpon* Reitter, 1889 exhibits the most pronounced troglomorphic characters among Coleoptera, and represents one of the most spectacular radiations of subterranean beetles. However, radiation, diversification, and biogeography of this genus have never been studied in a phylogenetic context. This study provides a comprehensive evolutionary analysis of the *Anthroherpon* radiation, using a dated molecular phylogeny as a framework for understanding *Anthroherpon* diversification, reconstructing the ancestral range, and exploring troglomorphic diversity. Based on 16 species and 22 subspecies, i.e. the majority of *Anthroherpon* diversity, we reconstructed the phylogeny using Bayesian analysis of six loci, both mitochondrial and nuclear, comprising a total of 4143 nucleotides. In parallel, a morphometric analysis was carried out with 79 landmarks on the body that were subjected to geometric morphometrics. We optimized morphometric features to phylogeny, in order to recognize the way troglomorphy was expressed in different clades of the tree, and did character evolution analyses. Finally, we reconstructed the ancestral range of the genus using BioGeoBEARS. Besides further elucidating the suprageneric classification of the East-Mediterranean Leptodirini, our main findings also show that *Anthroherpon* dates back to the Early Miocene (ca. 22 MYA) and that the genus diversified entirely underground. Biogeographic reconstruction of the ancestral range shows the origin of the genus in the area comprising three high mountains in western Montenegro, which is in the accordance with the available data on the paleogeography of the Balkan Peninsula. Character evolution analysis indicates that troglomorphic morphometric traits in *Anthroherpon* mostly evolve neutrally but may diverge adaptively under syntopic competition.

## Introduction

The subterranean environment—isolated, oligotrophic, and ecologically simpler than most other types of habitat—has long been considered as a “natural laboratory” for evolutionary studies, particularly for the study of speciation and processes of adaptation [1, 2]. Moreover, terrestrial cave animals are also excellent models for biogeographical studies since their present distribution tends to reflect those of ancestral surface ancestors [3, 4], given that subterranean dispersal is much more restricted. For these reasons, subterranean cave animals have figured prominently in studies about phenotypic adaptation, speciation, endemism, and evolutionary radiation [5–7]

Organisms inhabiting the subterranean environment evolve similar suites of morphological, physiological, and behavioural characteristics, known as troglomorphy or troglobiomorphy [8]. Troglomorphic characteristics include: eye degeneration, loss of wings (apterism), depigmentation, extreme development of sensory organs, longer life cycles, lower metabolic rate, and various body shape modifications [9–11]. A common body shape modification in cave-adapted arthropods is the increased length of antennae and legs compared to their surface relatives. The elongation of appendages has traditionally been explained as adaptation that facilitates effective foraging in oligotrophic cave habitat [12–14].

Also, cave fauna is well known for its extremely high degrees of short-range of endemism. Many troglobites have low vagility, and they are strongly and irreversibly adapted to cave conditions [9, 15–17]. This, plus the fact that cave systems are often fragmented and geologically isolated, has caused many cave organisms to be restricted to very small ranges. However, the relative importance of dispersal and vicariance in the biogeography of subterranean animals is still a matter of debate [18]. The most recent studies of cave beetles have shown that their present distribution could be chiefly due to ancient vicariance events [3, 4].

Until recently, the widely accepted view regarding the radiation of troglobites was that once a lineage has adapted to the subterranean environment within a karst unit, it is unable to expand or diversify over a larger area because of environmental constraints and, as a result, it remains restricted to a small geographical area [1, 5, 19]. The existence of widespread ancient troglobitic lineages was usually interpreted as the result of multiple independent colonisations followed by extinction of the ancestors [1, 19, 20]. Some studies support this hypothesis (for Pyrenean troglobitic Trechini and Leptodirini; [21, 22]), while others suggest a single subterranean colonization event [3, 4]. Still, the evolutionary dynamics and the origin of strictly subterranean lineages with multiple species, which could help resolve this debate, are understudied.

One group that has undergone extensive diversification in the subterranean environment are beetles of the tribe Leptodirini (Coleoptera: Leiodidae: Cholevinae), one of the largest groups of underground insects [3]. Leptodirini have a Palearctic distribution, with the highest diversity in the Mediterranean basin [23, 24]. To resolve their phylogeny, molecular approaches have been initiated in 1980 by Sbordoni [25]. Applying the technique of the molecular clock, Caccone & Sbordoni [26] gave a first estimation of a date of separation of the Sardinian from the Iberian-Pyrenean fauna. After this pioneering work, the next study on the molecular phylogenetics of Leptodirini was that of Ribera et al. [3]. The phylogeny of the Western Mediterranean species of Leptodirini including the Pyrenean fauna revealed that the main subterranean lineages became separated before the Early Oligocene [3].

**Fig 1:**
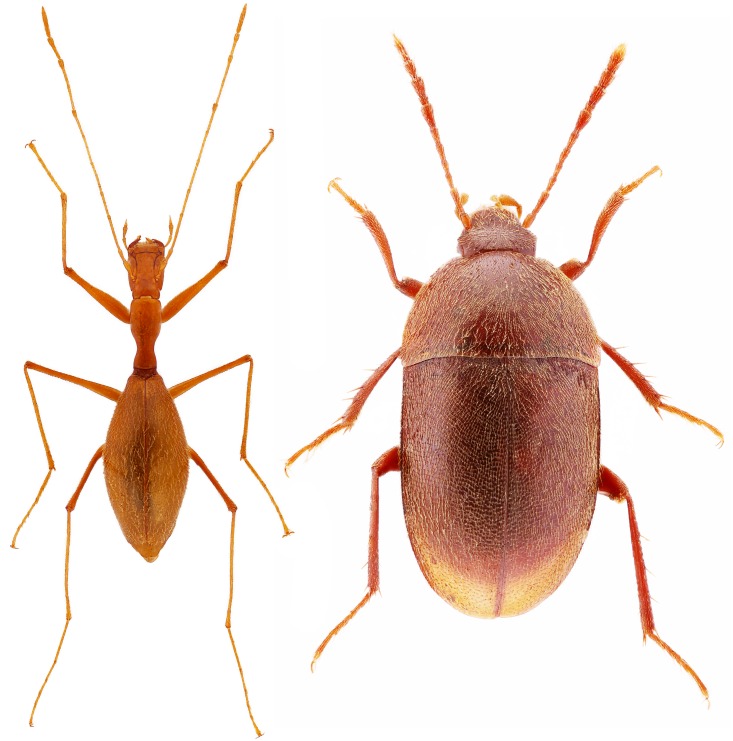
Habitus of *Anthroherpon*, the genus with the pronounced troglomorphic characters, in comparison to another subterranean genus *Sophrochaeta*. On the left: *Anthroherpon stenocephalum stenocephalum*, on the right: *Sophrochaeta oltenica densepunctata*.

These molecular studies focused on the western Mediterranean. Further east, the Dinaric Mountains have provided uninterrupted conditions for subterranean life for millions of years, and host a rich and diverse cave fauna, with complex, “hotspot-within-hotspot” patterns [27]. This mountain chain is recognized as having the world’s greatest species richness for the subterranean fauna [28–30]. The Leptodirini are the most species-rich group, comprising 175 species in 50 genera, most of which are endemic to the Dinarides [31]. Within Leptodirini, the subtribe Anthroherponina Jeannel, 1910 shows the most pronounced troglomorphic characters (Fig 1). Studies of these beetles have so far been based only on morphological characters [32, 33] and phylogenetic relationships inferred from morphology have not yet been tested with molecular data. The most species-rich genus of the subtribe is *Anthroherpon* Reitter, comprising 26 species and 55 subspecies, and showing some of the most remarkable radiations among all Leptodirini. All species of this genus have developed typical troglomorphic modifications such as complete anophthalmy and apterism, and they exhibit to various degrees: extreme elongation of appendages, head, pronotum, and pseudo-physogastry (the abdomen is greatly enlarged and appears to be swollen due to an empty space between the elytra and the thin abdominal membrane). These troglomorphic adaptations are not linked to different ecological requirements, and clearly need to be studied in a phylogenetic framework, to better understand how they developed during evolution.

We provide here a comprehensive evolutionary analysis of the *Anthroherpon* radiation, using a dated molecular phylogeny as framework for understanding *Anthroherpon* diversification, reconstructing its ancestral range, and exploring troglomorphic diversity. Our study represents the first comprehensive analysis of Dinaric terrestrial troglobites that involves a dated phylogeny, ancestral range reconstruction, and morphometric approaches.

## Materials and methods

### Multi-locus molecular phylogenetics

#### Taxon sampling

Specimens were collected in caves and sinkholes (up to 400 m deep) of the Dinaric range, in Montenegro, and Bosnia and Herzegovina, with a focus on the type localities (S3 Table). In Montenegro, specimens were collected under permit 02-UPI-608/1 issued by the Environment Protection Agency of Montenegro; in Bosnia and Herzegovina, material was collected within the project “Protection of underground biodiversity in the Neretva River catchment area—Identifying and raising the awareness of conservation hotspots” implemented by Centar za krš i speleologiju, Sarajevo (no special permit was required); municipality of Ravno (Bosnia and Herzegovina) gave us permission to collect in Vjeternica. In total, we sampled 45 species of Leptodirini belonging to 12 genera, among which 16 species and 23 subspecies of the genus *Anthroherpon*, covering its entire distribution range. For *Anthroherpon*, two individuals per population were used for amplification and sequencing. Because we wanted to date the divergence of *Anthroherpon* it was necessary to expand the outgroup to include taxa for which the age of divergence is known. Therefore, we included 57 species of Leptodirini from Ribera et al. [3] for which the same five genetic markers were available (only COI Folmer was not available for those 57 taxa). We chose the same outgroup consisting of 8 species belonging to different tribes of Cholevinae (Anemadini, Ptomaphagini, Cholevini): *Ptomaphagus tenuicornis*, *Ptomaphagus troglodytes*, *Nargus algiricus*, *Nargus velox*, *Speonemadus angusticollis*, *Catops fuliginosus*, and *Catops tristis*, and *Agathidium* sp. as a member of the phylogenetically separated subfamily Leiodinae. To root the tree, we used *Agathidium* from the subfamily Leiodinae. The final data matrix comprised 4143 bp of 113 species from 51 genera.

#### DNA extraction, PCR amplification, and sequencing

The specimens used in the study were collected alive and preserved in 96% ethanol in the field. DNA was extracted from whole specimens or from one leg with a standard phenol–chloroform extraction [34] or the DNeasy Tissue Kit (Qiagen GmbH, Hilden, Germany). Voucher specimens are stored in CINJ and CNHM, and DNA aliquots are kept in the tissue collections of Naturalis Biodiversity Center.

We amplified six fragments of five genes, three mitochondrial and two nuclear: (i) two non-overlapping sections of mitochondrial cytochrome c oxidase subunit 1, the 5’ and 3’ halves, which we here term COIa and COIb, respectively; (ii) the 5’ end of the mitochondrial large ribosomal unit plus the Leucine transfer RNA plus the 3’ end of NADH dehydrogenase subunit 1 (rrnL-nad1); (iii) an internal fragment of cytochrome b (cob); (iv) the 5’ end of the small ribosomal unit, 18S rRNA (SSU); (v) an internal fragment of the large ribosomal unit, 28S rRNA (LSU). Primers used are given in Table 1. Each 25 μl PCR mixture included 1 μl (10 pmol) of each primer, 2.5 μl 10x PCR buffer, 0.5 μl dNTPs, 0.25 μl Taq-polymerase, 18.8 μl ddH2O and 5 μl template DNA. PCR cycles were run at the following conditions: 3 min at 94 °C, followed by 40 cycles of 15 s at 94 °C, 30 s at 54 °C and 40 s at 72 °C, and finally, 5 min at 72 °C. PCR fragments were sequenced directly and sequencing was performed by BaseClear (www.baseclear.com). Sequences were assembled and edited using Geneious version 8.0.5 [35]. DNA sequences obtained for each genetic marker were aligned separately using MAFFT version 7 [36].

**Table 1:** Primers used in the study.

Fragment	Name	Sense	Sequence 5’-3’	Reference
*COIa*	LCOI-1490	F	GGTCAACAAATCATAAAGATATTG	Folmer et al. (1994)
*COIa*	HCOI-2198	R	TAAACTTCAGGGTGACCAAAAAATCA	Folmer et al. (1994)
*COIb*	Jerry	F	CAACATTTATTTTGATTTTTTGG	Simon et al. (1994)
*COIb*	Pat	R	TCCAATGCACTAATCTGCCATATTA	Simon et al. (1994)
*cob*	CB3	F	GAGGAGCAACTGTAATTACTAA	Barraclough et al. (1999)
*cob*	CB4	R	AAAAGAAA(AG)TATCATTCAGGTTGAAT	Barraclough et al. (1999)
*rrnL-nad1*	16Sbi F	F	ACATGATCTGAGTTCAAACCGG	Simon et al. (1994)
*rrnL-nad1*	FawND1 R	R	TAGAATTAGAAGATCAACCAGC	Simon et al. (1994)
*SSU*	5’	F	GACAACCTGGTTGATCCTGCCAGT	Shull et al. (2001)
*SSU*	b5.0	R	TAACCGCAACAACTTTAAT	Shull et al. (2001)
*LSU*	Ka	F	ACACGGACCAAGGAGTCTAGCATG	Ribera et al. (2010)
*LSU*	Kb	R	CGTCCTGCTGTCTTAAGTTAC	Ribera et al. (2010)

#### Phylogenetic analysis

DNA sequences from the current study (S1 Table) and a selection of data from Ribera et al. [3] were combined using Geneious v9.1.2 [35] as a workbench. For each genetic marker, an alignment was made using MUSCLE [37]. For LSU and SSU, in some regions, alignment was ambiguous due to large indels; these regions were removed from the final data matrix. Translations of coding genes were checked for consistency and validity. We have refrained from nucleotide substitution model fitting for two reasons. First, model-fitting software such as jModelTest [38] takes an expected initial tree against which the software fits the model, and that model is then used to fit the phylogenetic reconstruction. This may introduce a degree of circularity in the procedure. Second, our method of choosing a general model allows the Bayesian MCMC algorithm to fit the model and the tree at the same time, which in our opinion is a more objective method.

Prior to the analysis in BEAST, we performed Bayesian phylogenetic analyses on the entire dataset, to clarify the taxonomic status of all taxa included in the analyses. The analysis was conducted using MrBayes 3.2.2 [39] on the CIPRES web portal [40], while data for all markers were linked and the most general substitution model (gamma, with all substitutions possible) was set. Two replicates of 10 x 10^6^ generations each were run, sampling values and trees every 5000 generations. Convergence diagnostics were run using Tracer version 1.5 [41], where ESS values for all parameters were >>200. After discarding a 25% burn-in, the resulting majority-rule consensus tree was visualized using FigTree version 1.4 [42]. Genetic distances among subspecies in three *Anthroherpon* species (*A*. *taxi*, *A*. *hoermanni*, *A*. *matzenaueri*) were high (genetic distance in barcoding region >3%), meaning that these may be used as species level units in the BEAST analysis. For this, the specimen-level phylogenies were pruned so that a single operational taxonomic unit (OTU) remained per species.

Phylogenetic analysis was performed in BEAST2 v2.3.2 [43] on the CIPRES web portal [40], using a single individual per species. Site models for all six sequence alignments were unlinked (i.e. six partitions), with the most general GTR model selected for each partition. A relaxed lognormal clock was chosen, where all partitions were linked together. The Yule model was chosen for the tree prior. The analysis was run twice to check for consistency, each time with a chain length of 100 million generations, sampled and stored every 10 000 generations. The rest of the analysis was done like in Bayesian approach. To visualise the (in)congruence between the phylogenetic signal in nDNA versus mtDNA, we used the web-based software http://Phylo.io [44].

Reliable dating for vicariant events or the age of subterranean habitats of the Dinaric Mountains has not yet been established [45]. Ribera et al. [3] used the separation of the Sardinian microplates from mainland Europe to calibrate the phylogeny of Western Mediterranean Leptodirini. In line with their findings, we calibrated our phylogeny by setting an expected age of 37.8MY for the sister clade of the Sardinian taxa (“Western Mediterranean Leptodirini”). To this end, we defined a calibration prior on the clade of “Western Mediterranean Leptodirini” with a normal distribution (mean of 37.8 MY, standard deviation of 2 MY) in our BEAST2 run.

To get an impression of relative branch lengths and potential long branch attraction, we also conducted maximum likelihood analysis. Maximum likelihood searches were performed with RAxML-HPC BlackBox (v8.2.10) [46] on the Cipres Science Gateway [47], which uses maximum likelihood-based inference of large phylogenies to simultaneously find the topology and branch lengths. We used a partitioned, general GTR+I+Γ model, with partitions set to agree with the different genetic markers as used with our Bayesian analysis. Otherwise, we used default settings. Support was measured with 1,000 bootstrap replicates. We used *Agathidium* sp. (sample MNCN_AI1305) as an outgroup.

### Reconstruction of the ancestral range

The ancestral geographic range of *Anthroherpon* was inferred using the R package BioGeoBEARS 0.2.1 [48, 49]. This model approach directly tests the fit of commonly used biogeographical inference models: dispersal-extinction-cladogenesis model (DEC) [50], maximum likelihood versions of dispersal-vicariance analysis (DIVALIKE) [51], and Bayesian biogeographical inference (BAYAREALIKE) [52]. The ultrametric tree from the Bayesian relaxed molecular clock analysis, which includes one member per (sub)species, was used as input tree. We identified 16 different geographic areas, corresponding to mountain ranges or caves, inhabited by *Anthroherpon* species. We could not include a larger number of areas due to computational limitations. Each species was coded as being present or absent in each of these areas (Table 2), and a maximum number of areas occupied by a single species was set to 3. For a detailed list of the localities see S1 Table. For all three models, we compared the fit with and without a founder event parameter, “j”, which describes a speciation event where a “jump dispersal” event quickly results in an evolutionarily independent lineage [49]. The “j” parameter allows for a daughter lineage to immediately occupy via long-distance dispersal a new area that is different from the parental lineage (ABCD > ABCD, E). Finally, we tested a novel distance-based dispersal model (+x) where dispersal probability is multiplied by distance to the power “x” [53]. For this purpose, we created a matrix indicating distances between selected geographical areas (S2 Table). Distances between the area centroids were measured in kilometres on Google Earth. These distances were used in the constrained distance-dependent dispersal matrix. In total, we implemented 12 models in BioGeoBEARS. The models were compared to each other using two different methods: (1) the Likelihood of all models were compared to each other with Akaike Information Criterion (AIC). This was done in two blocks: all the models without "x" compared to each other, and all the models with "x" compared to each other; (2) the nested models were compared with each other using a chi-squared test. This was mainly to determine if models with "j" parameter are preferred or not.

**Table 2:** List of taxa and their geographic distributions, as included in the biogeographic analysis. Abbreviations of geographic areas as follows: A. Golubovića pećina; B. Mravinjac; C. Zelengora; D. Lebršnik; E. Velež; F. Dobreljica; G. Moračke planine; H. Sinjajevina; I. Tebević +Jahorina; J. Bjelašnica; K. Kečina stena; L. Banja pećina; M. Durmitor, N. Orjen; O. Prokletije, P. Županska pećina. Details of the localities are given in S1 Table.

Areas in Figs 3 and 4.	A	B	C	D	E	F	G	H	I	J	K	L	M	N	O	P
*A*.*cylindricolle cylindricolle*	1	0	0	0	0	0	0	0	0	0	0	0	0	0	0	0
*A*.*primitivum jeanneli*	0	1	0	0	0	0	0	0	0	0	0	0	0	0	0	0
*A*.*hoermanni hoermanni*	0	0	1	0	0	0	0	0	0	0	0	0	0	0	0	0
*A*.*hoermanni sericeum*	0	0	1	0	0	0	0	0	0	0	0	0	0	0	0	0
*A*.*hoermanni hypsophilum*	0	0	0	1	0	0	0	0	0	0	0	0	0	0	0	0
*A*.*hoermanni orlovacensis*	0	0	0	1	0	0	0	0	0	0	0	0	0	0	0	0
*A*.*ganglbaueri ganglbaueri*	0	0	0	0	1	0	0	0	0	0	0	0	0	0	0	0
*A*.*matulici*	0	0	0	0	0	0	0	0	0	0	0	0	0	1	0	0
*A*.*matzenaueri matzenaueri*	0	0	0	0	0	0	0	0	0	0	0	0	0	0	0	0
*A*.*matzenaueri taliensis*	0	0	0	0	0	0	1	0	0	0	0	0	0	0	0	0
*A*. *sinjajevina*	0	0	0	0	0	0	0	1	0	0	0	0	0	0	0	0
*A*.*charon*	0	0	0	0	0	0	0	0	1	0	0	0	0	0	0	0
*A*.*erebus scheibeli*	0	0	0	0	0	0	0	0	1	0	0	0	0	0	0	0
*A*.*pygmaeum stricticolle*	0	0	0	0	0	0	0	0	0	1	0	0	0	0	0	0
*A*.*harbichi*	0	0	0	0	0	0	0	0	0	0	1	0	0	0	0	0
*A*.*weiratheri*	0	0	0	0	0	0	0	0	0	0	1	0	0	0	0	0
*A*.*stenocephalum stenocephalum*	0	0	0	0	0	0	0	0	0	0	0	1	0	0	0	0
*A*.*zariquieyi*	0	0	0	0	0	0	0	0	0	0	0	0	1	0	0	0
*A*.*latipenne*	0	0	0	0	0	1	1	0	0	0	0	0	0	1	0	0
*A*.*t*. *taxi* + A. *t*. s*ydowi*	0	0	0	0	0	0	1	0	0	0	0	0	0	1	0	0
*A*.*taxi albanicum*	0	0	0	0	0	0	0	0	0	0	0	0	0	0	1	0
*A*.*taxi remyi*	0	0	0	0	0	0	0	0	0	0	0	0	0	0	0	1

### Linear morphometrics and three-dimensional geometric morphometrics

#### Data collection

Specimens of the genus *Anthroherpon* were pin-mounted on a rectangular piece cardboard affixed to the ventral side of the body and labelled. A total of 102 specimens belonging to 16 species and 20 subspecies of *Anthroherpon* were measured for morphometric analyses (S3 Table). All of these species except *A*. *matulici* were included in the phylogenetic analysis. Specimens are stored at the CINJ and at the MNHN. To exclude sex-specific characters (males have one additional protarsomere), measurements data were only collected from male beetles.

Linear and 3D morphometric measurements were obtained with a Micro-Vu Vertex 251HC (https://www.microvu.com/, St. Wendel, Germany), three-dimensional set-up, using Inspec Metrology Software (https://www.inspec-inc.com/, Canton, MI, USA). In total, we recorded 79 landmarks (S1 Fig) on 152 specimens. Linear measurements were taken on antennae, maxillary palps, head, pronotum, elytra, and legs, using 53 landmarks. Shape analyses were based on 40 landmarks: 6 on the head, 18 on the pronotum, and 16 on the elytra. Each individual was measured three times. In a small number of cases, obvious measurement errors were detected a posteriori (values differing by a factor of >2 from the other two replicates of the same individual). /The full list of measured material is given in S3 Table and the list of morphometric data is given in S4 Table.

#### Analysis of morphometric data

To compare the shapes of the different individuals, the 3D coordinates were first superimposed for each body part using a Generalized Procrustes Superimposition (GPA) [54]. Because the three shapes present an axis of symmetry, they were symmetrized during the superimposition to remove the asymmetric variation [55]. The resulting aligned coordinates were then projected from the raw shape space to the tangent shape space before using them as variables to quantify the shapes. The GPA and subsequent analyses of shapes and linear measurements were performed in R (R development core team, 2016), with the packages “ape”, “geomorph”, and “phytools” [56–58].

1. Exploration of troglomorphic diversity within *Anthroherpon*
  Degree of troglomorphy in arthropods is often evaluated on the basis of relative elongation of the appendages (maxillary palps, antennae, legs) [8]. To quantify the degree of troglomorphy within *Anthroherpon* based on these proxies, we compared the lengths of these appendages and their segments relative to body length (measured from the posterior margin of the clypeus to the apex of the elytra) between species. We also studied the evolution of body and appendage lengths in the *Anthroherpon* clade by estimating the ancestral states of the segments’ lengths assuming a Brownian motion model of evolution [59], function ‘fastanc’ of Phytools, [58] and by testing their phylogenetic signal [60].
2. Exploration of shape evolution within *Anthroherpon*
  To quantify the habitus shape variation within the genus *Anthroherpon* (s. str.), we analyzed shapes of the three main body parts (head, pronotum, and elytra), using geometric morphometrics. For each body part, each species’ or subspecies’ shape was estimated by the shape of the specimen closest to the mathematical mean shape of the taxon. The data were subjected to character evolution analyses by testing for phylogenetic signal in each of the three body part datasets separately, using the K of Blomberg [60, 61]. Tests were performed using 10 000 permutations, and a risk alpha of 5%. We then used our phylogeny to retrace the shape evolution in the phylomorphospace using ancestral state reconstruction according to a Brownian motion model of evolution [62].
3. Character divergence in two syntopic species, *A*. *harbichi* and *A*. *weiratheri*
  Since *A*. *harbichi* and *A*. *weiratheri* are two sister species living in syntopy, we aimed at testing whether their speciation may have been accompanied by morphological divergence between them greater than expected under evolutionary drift. To test this hypothesis, we compared the evolutionary rates of these two species with those of the rest of the tree [63].

## Results

### Genus-level phylogeny of Leptodirini

**Fig 2:**
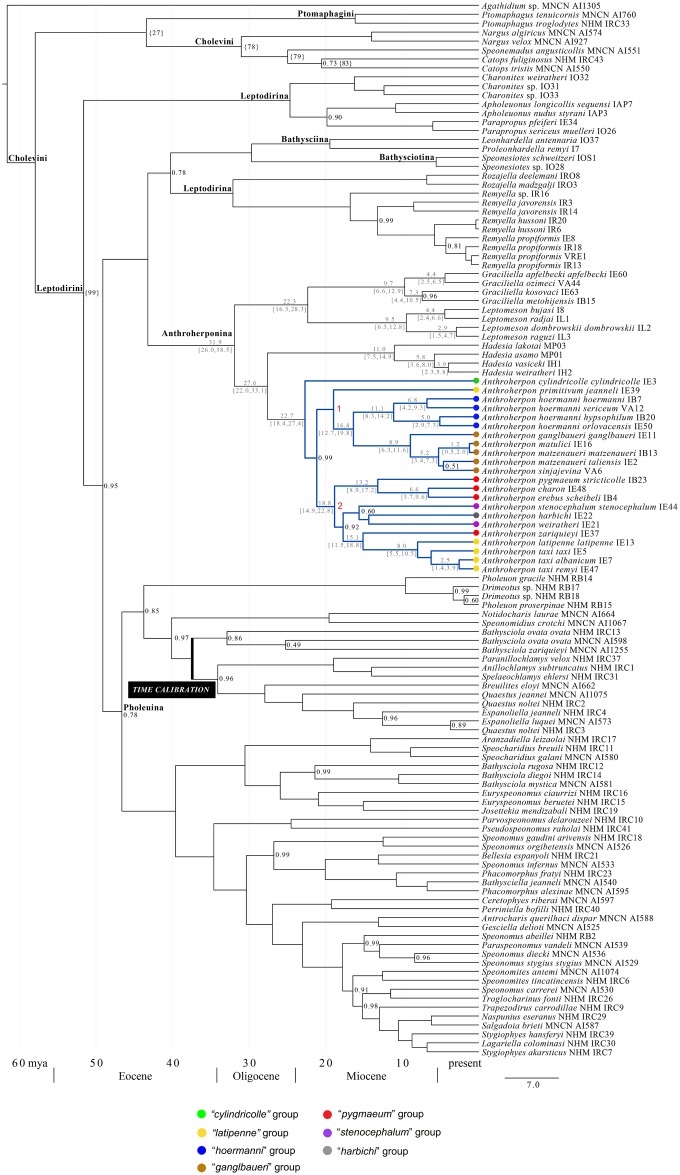
Ultrametric tree of the phylogeny of the genus *Anthroherpon* obtained with Beast using as calibration the separation of *Bathysciola zariquieyi* from its sister obtained by Ribera et al. [3]. Numbers above nodes, estimated age (in MYA); every node supported by less than 100% has the support value shown. Numbers 1 and 2 in red indicate two major clades of *Anthroherpon*.

Our phylogenetic analyses (Fig 2) revealed that the subtribe Leptodirina is polyphyletic: three genera of this subtribe (*Charonites* Apfelbeck, 1907, *Apholeuonus* Reitter, 1889, and *Parapropus* Ganglbauer, 1899) form a highly-supported clade which is a sister clade to other members of the tribe Leptodirini, while two genera previously tentatively placed in Leptodirina (*Remyella* Jeannel, 1931 and *Rozajella* S. Ćurčić, Brajković & B. Ćurčić, 2007) [64] form a clade with Bathysciina+Bathysciotina. The latter clade is weakly supported (posterior probability 0.78) and also unlikely on morphological grounds. The origin of the clade comprising *Rozajella* and *Remyella* was estimated to have occurred in the Early Oligocene (ca. 32 MYA) while “true” Leptodirina (*Charonites*, *Apholeuonus*, and *Parapropus*) are of more recent age: Late Oligocene (ca. 25 MYA).

The study confirmed the monophyletic origin of the analysed taxa of the subtribe Anthroherponina (Fig 2), which can be dated to the Early Oligocene (ca. 32 MYA). The origins of the four analysed genera of this subtribe (*Graciliella* Njunjić, Perreau, Hendriks, Schilthuizen & Deharveng, 2016, *Leptomeson* Jeannel, 1924, *Hadesia* Müller, 1911, and *Anthroherpon*) were estimated to a relatively narrow time window in the Late Oligocene—Early Miocene. Two major sister clades can be recognized: one comprising the genera *Graciliella* and *Leptomeson* and one comprising *Anthroherpon* and *Hadesia*. The separation of *Graciliella* and *Leptomeson* was estimated to have taken place in the Early Miocene (ca. 22 MYA), while *Anthroherpon* and *Hadesia* split earlier, in the Late Oligocene (ca. 27 MYA). The phylo.io analysis shows that within the Anthroherponina, the genus-level topologies based on nDNA and mtDNA separately are highly similar, but that dissimilarities occur in the deeper intrageneric topology for *Remyella* (S2 Fig).

The phylogram resulting from the maximum likelihood analysis (S3 Fig) shows similar branch lengths overall with the possible exception of *Charonites sp*. IO33 and *Bathysciola_ovata_ovata*_MNCN_AI598. Within *Anthroherpon* (see next section), no long branches are noticeable.

### Phylogeny of the genus *Anthroherpon*

The phylogenetic analysis shows the monophyletic origin of the genus *Anthroherpon*, which was estimated to have started to diverge in the Early Miocene (ca. 22 MYA) (Fig 2). The most basal clade that branches off contains a single species: *A*. *cylindricolle s*. *str*. The genus is otherwise split into two main, highly supported (pp 100%) clades defined by nodes 1 and 2. Both clades started to diverge approximately around the same time (ca. 19 MYA) in the Early Miocene. The clade defined by node 1 is composed of two monophyletic clades comprising species of the “*hoermanni*” and “*ganglbaueri*” species group, and the single species *A*. *primitivum jeanneli*, which forms a separate clade. All three subclades within the clade defined by node 1 are well-supported (pp 100%). The clade defined by node 2 contains three main clades: one highly supported clade (pp 100%) comprising three species of the “*pygmaeum*” species group, and two sister clades containing the rest of *Anthroherpon* species belonging to the “*pygmaeum*”, “*harbichi*”, “*stenocephalum*”, and “*latipenne*” species groups.

These generaly high levels of support are reached despite the fact that phylo.io analysis shows some intrageneric dissimilarity in mitochondrial versus nuclear DNA signals (S2 Fig).

### Ancestral range reconstruction

**Fig 3:**
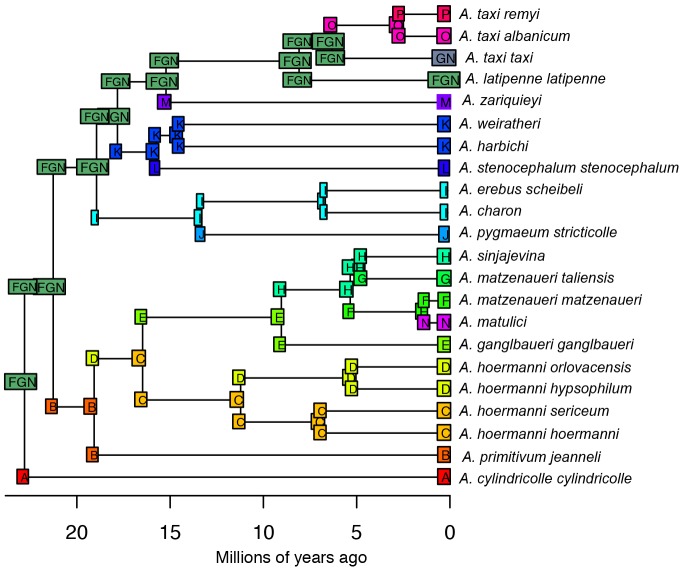
Preferred model of biogeographic reconstruction (BAYAREALIKE+*j*), according to biogeographic analysis of *Anthroherpon* distribution. Abbreviations of geographic areas as follows: A. Golubovića pećina; B. Mravinjac; C. Zelengora; D. Lebršnik.; E. Velež.; F. Dobreljica.; G. Moračke planine; H. Sinjajevina; I. Tebević+Jahorina; J. Bjelašnica.; K. Kečina stena; L. Banja pećina; M. Durmitor, N. Orjen; O. Prokletije, P. Županska pećina. *A*. *taxi taxi* also includes *A*. *taxi sydowi* as indicated in the Table 2.

**Fig 4:**
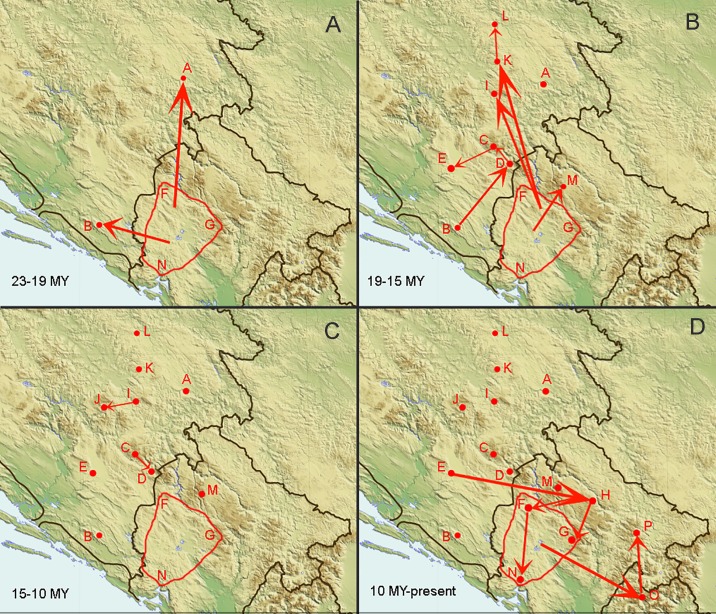
Inferred biogeographic history of *Anthroherpon* using the BAYAREALIKE+*j* model. Geographical areas used in the analyses are marked with same letters as in Fig 3. Arrows denote dispersal directions.

The results of ML inference of each biogeographical model are given in Tables 3 and 4. Pairwise likelihood ratio test detected a significantly (p < 0.05) better fit of models with a founder effect (j) (results not shown). Model comparisons based on AIC consistently favoured the BAYAREALIKE model with a founder effect (j), either with or without the distances between the areas taken into account. Under this model the most likely ancestral range of the *Anthroherpon* clade consists of the areas F, G, and N (Dobreljica, Moračke planine, and Orjen) (Figs 3 and 4). From this ancestral range, several single dispersal events into areas A, B, I, K, M, and O occurred. Subsequent dispersal events from these areas explains the occurrence of *Anthroherpon* in the remaining areas.

**Table 3:** BioGeoBEARS results for the genus *Anthroherpon* based on BEAST topology. Models tested without the distances between the areas taken into account.

Model	LnL	Degrees of freedom	d	e	j	AIC	AIC wt
BAYAREALIKE+j	-63.06	3	1.0e-07	0.0031	0.10	132.1	0.98
BAYAREALIKE	-94.97	2	0.0079	0.11	0	193.9	3.7e-14
DIVALIKE+j	-67.13	3	0.0009	1.0e-12	0.087	140.3	0.017
DIVALIKE	-85.67	2	0.0044	0.0094	0	175.3	4.0e-10
DEC+j	-68.59	3	0.0008	1.0e-12	0.07	143.2	0.0039
DEC	-90.7	2	0.0057	0.043	0	185.4	2.6e-12

**Table 4:** BioGeoBEARS results for the genus *Anthroherpon* based on BEAST topology. Models tested with the distances between the areas (+x) taken into account.

Model +x	LnL	Degrees of freedom	d	e	x	j	AIC	AIC wt
BAYAREALIKE+j	-63.94	4	1.0e-07	0.0029	0.086	0.11	135.9	0.96
BAYAREALIKE	-93.75	3	0.05	0.11	-0.43	0	193.5	2.9e-13
DIVALIKE+j	-67.39	4	0.0011	1.0e-12	-0.0030	0.064	142.8	0.03
DIVALIKE	-84.99	3	0.0079	0.014	-0.13	0	176	1.9e-09
DEC+j	-68.48	4	0.0007	1.0e-12	0.029	0.081	145	0.010
DEC	-90.64	3	0.0061	0.044	-0.014	0	187.3	6.6e-12

### Results of morphometric analyses

Although the general elongation of extremities is a well-known aspect of troglomorphy, we find that in *Anthroherpon*, different types of extremities show different patterns. Whereas leg elongation and antenna elongation are strongly correlated among species (*r* = 0.929, *p* = 2.575x10^-8^), maxillary palps elongation is independent of these (no significant correlations with either leg or antenna length). As a result, the group of species with the longest legs and antennae, relative to the body length (i.e., *A*. *hoermanni*, *A*. *pygmaeum*, *A*. *harbichi*, *A*. *latipenne*, *A*. *taxi*) does not overlap with the group of species with the greatest relative maxillary palps length (i.e., *A*. *cylindricolle*, *A*. *ganglbaueri*, and *A*. *zariquieyi*).

**Fig 5:**
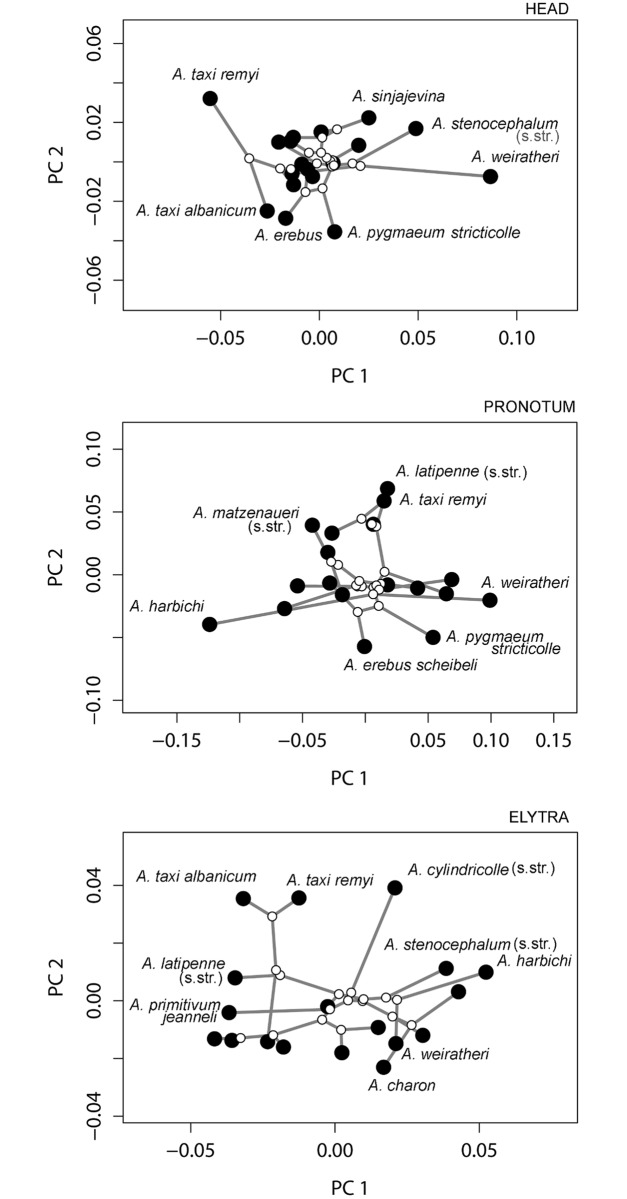
Phylomorphospaces for the genus *Anthroherpon*, shown for each body part separately. Open symbols are internal nodes; filled symbols are terminal nodes. Only the two first principal components of a principal component analysis of each shape are depicted.

We found the following values for Blomberg’s *K*. Head: *K* = 0.57 (not significant, *P* = 0.3924); Pronotum: *K* = 0.65 (significant, *P* = 0.0014); Elytra: *K* = 0.75 (significant, P = 0.003). The two first dimensions of the corresponding phylomorphospaces are shown in Fig 5. These suggest that most species present a similar head shape, except for *A*. *stenocephalum*, *A*. *weiratheri*, and *A*. *taxi remyi*; pronotum shape appears to vary homogeneously, with the exception of *A*. *weiratheri* and *A*. *harbichi* (see below); elytra shape variation also seems relatively homogeneous in the phylomorphospace, with no apparent trend in shape variation except for the influence of the phylogeny. We should add the caveat, though, that the first two principal components explain 51.3%, 34.6%, and 40.6% of the relative size variation for the head, pronotum, and elytra, respectively. Additional components of variability may deviate from the phylomerphospace networks of Fig 5.

**Fig 6:**
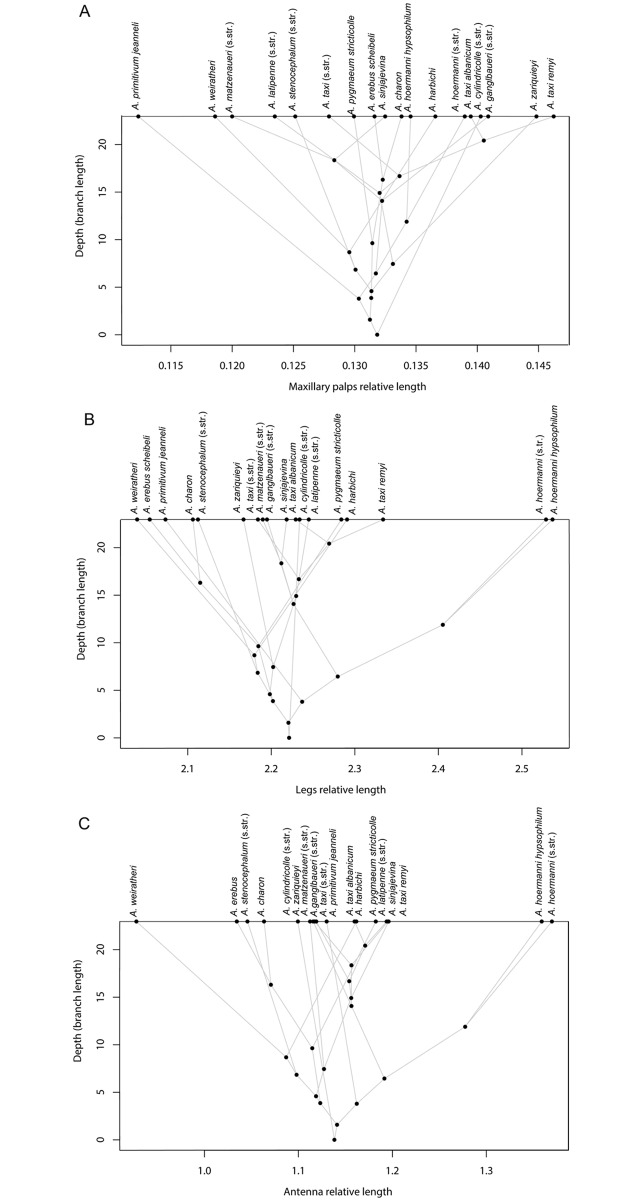
Evolution of the lengths of the appendages (completeness sake, the genus *Graciliella* is also included). A. Maxillary palps relative length; B. Legs relative length; C. Antenna relative length.

The evolution of the relative lengths of antennae and legs mainly follows the phylogeny (Fig 5B and 5C) with significant phylogenetic signals (Antenna: K = 1.056, p = 0.004; Leg: K = 0.979, p = 0.02). The evolution of maxillary palps shows a different pattern (Fig 6A), with many branches crossing each other and no significant phylogenetic signal (K = 0.614, p = 0.33).

**Fig 7:**
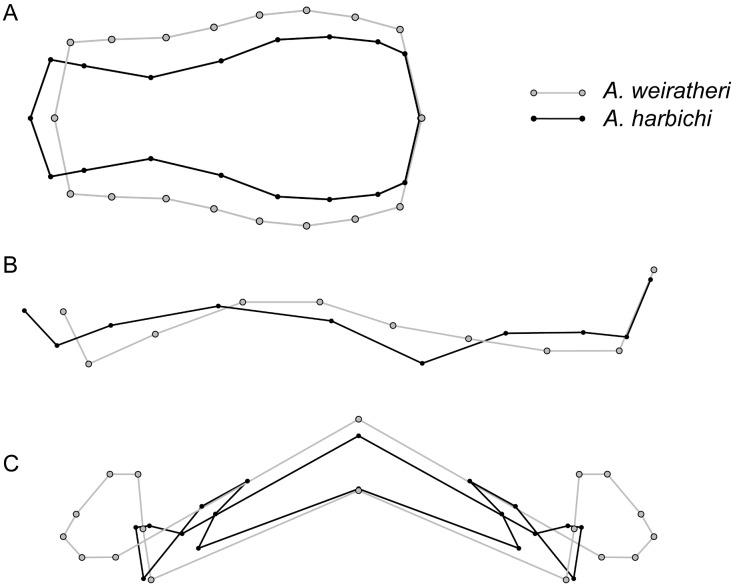
Non-amplified mean differences in pronotum shape between *A*. *weiratheri* and *A*. *harbichi*, shown in dorsal (A), lateral (B), and frontal (C) view.

The evolutionary rates found in the two syntopic species, *A*. *harbichi* & *A*. *weiratheri*, were significantly different for the pronotum (2.6 times higher; *P* = 0.014; see Fig 7). All other body parts (except relative maxillary palps length; see Fig 6A) showed morphological divergence rates in line with that in the rest of the genus.

## Discussion

Although the genus *Anthroherpon* is the focus of this paper, our results also show the polyphyly of the subtribe Leptodirina, suggesting that the tribal assignation of *Remyella* Jeannel, 1931, *Rozajella* S. Ćurčić, Brajković & B. Ćurčić, 2007 and also *Nonveilleriella* Perreau & Pavićević, 2008 should be reconsidered. However, the clarification of this question requires to take into consideration the whole tribe Leptodirina and not only the small number of genera used in this work (*Apholeuonus* Reitter, 1889, *Charonites* Apfelbeck, 1907, and *Parapropus* Ganglbauer, 1899). Enlarging the scope of future phylogenetic analyses around *Anthroherpon* would allow better understanding important evolutionary and biogeographical questions. That would be another step towards a complete phylogeny of Cholevinae.

The dated phylogeny we reconstructed for *Anthroherpon* and related genera (Fig 2), shows that the large genus *Anthroherpon* began to diverge approximately in the early Miocene (22 MYA), ca. 5 million years after breaking away from a lineage to a divergent, hygropetric genus also endemic of the Dinarides, *Hadesia*. Although we did not do a formal lineages-through-time analysis, the branching points within *Anthroherpon* are spaced quite evenly through time, suggesting that the evolutionary radiation of the genus has not been marked by any major changes in diversification rate.

Even when disregarding the polyphyly of *Anthroherpon* that necessitated the erection of the new genus, *Graciliella* [65], our results only partly support the traditional, morphology-based, subdivision of the genus *Anthroherpon* into 7 species groups [32, 66]. Our phylogenetic reconstruction shows that only the “*ganglbaueri*” and “*cylindricolle*” species groups are monophyletic, while all others show polyphyly.

**Fig 8:**
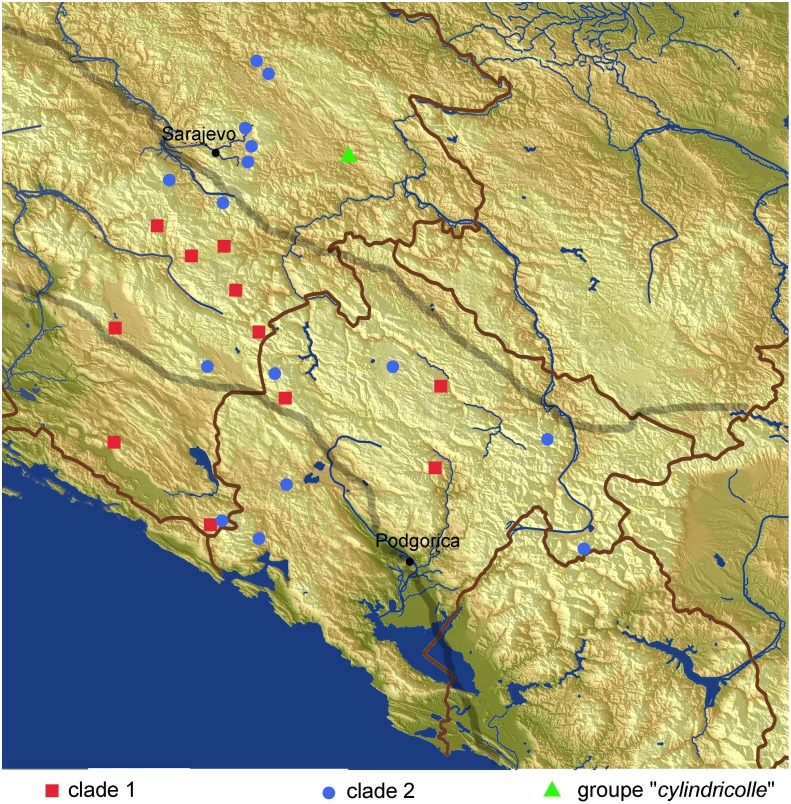
Distribution of *Anthroherpon* species included in the phylogenetic reconstruction obtained with BEAST (Fig 2). Different colours and symbols denote main clades of the phylogenetic tree. Thick grey lines show delimitation of the Dinaric karst in three main belts, from the southwest to northeast: the Low coastal Adriatic karst, the High mountain karst, and the Low continental interior karst [67].

The phylogenetic reconstruction shows a certain degree of geographic structuring (Fig 8) regarding the three main geomorphological units of the Dinaric karst (three belts parallel to the Adriatic Sea: Adriatic karst, the High mountain karst, and the Low continental interior karst [67]). Namely, the clade defined by node 1 chiefly contains the “*hoermanni*” species group and the “*ganglbaueri*” species group, distributed in the High mountain karst (the only exceptions are *A*. *matulici* and *A*. *primitivum jeanneli* from the Low coastal Adriatic karst). In contrast, the clade defined by node 2 contains species from very different parts of the range, stretching through all three belts of the Dinaric karst.

This geographic structuring allows formal and informal analyses of the biogeographic history. Our formal reconstruction of the biogeographic history shows four successive phases (Fig 4), characterized by: (i) an origin in western Montenegro and dispersal of the common ancestor of *Anthroherpon* into eastern Bosnia and Herzegovina; (ii) further, multiple movements through western Montenegro and eastern Bosnia and Herzegovina, mostly towards the north and northeast; (iii) a period of dispersal stagnation; (iv) further dispersals, mostly in a south-eastern direction, into eastern Montenegro and Albania. Dispersal events occurred mostly during the Miocene, when the mountain ranges within the Dinarides already had the present spatial distribution [68, 69]. We therefore may assume that the large-scale tectonic events that formed these mountain ranges preceded the *Anthroherpon* radiation.

Our biogeographic reconstruction of the ancestral range (Figs 3 and 4) shows the origin of the genus in the area comprising three high mountains in western Montenegro: Orjen (1894 m), Dobreljica (1834 m), and Moračke planine (2226 m). From this area the presumed *Anthroherpon* ancestor dispersed to the other parts of its present range. The origin of the genus in western Montenegro is in accordance with the available data on the paleogeography of the Balkan Peninsula. In the Lower Miocene, the northeastern part of the Dinarides was covered by the Dinaric Lake System that extended north to Lower Austria [70, 71]. Western and southern Montenegro were not covered by water during that time, which is consistent with an initiation of *Anthroherpon* radiation there. During the Middle and Late Miocene and the Pliocene lakes remained only in the depressions [72, 73] which may have limited dispersal routes to the dry areas in between.

Within *Anthroherpon*, it appears that for body shape (except head shape, which is more conserved) K-values are close to 1.0 and display significant phylogenetic signal. This suggests that in most species, presumably after isolation in allopatry, body shape evolves slowly following a Brownian motion model: body shape differences between species are roughly proportional to their phylogenetic distance and there is no strong divergence (K>1.0) or stasis (K<1.0).

Where troglomorphy in the appendages is concerned, we see that some species have more strongly elongated antennae and legs relative to their body size (Fig 6B and 6C). These proportions follow the phylogeny, which may mean that, within this entirely subterranean group, differences in elongation are neutral and not the result of rapid adaptation. If the latter were the case, we would expect to see many branches crossing each other in the phylomorphospace, which is not the case.

However, there are a few indications that, under certain circumstances, morphometric traits may evolve adaptively. Firstly, in contrast with the elongation of the other appendages (i.e. antenna and legs), the evolution of maxillary palps length relative to the body length is not reflecting the phylogeny (Fig 6A)Hope. Since maxillary palps are directly involved with food uptake [74], this may mean that this morphometric character evolves under the influence of functional constraints linked to nutrition. Some species (*A*. *primitivum*, *A*. *weiratheri*) evolved relatively short maxillary palps compared with their sister species, whereas others (*A*. *zariquieyi*, *A*. *taxi remyi*) evolved much longer maxillary palps. No link with environmental parameters has been detected so far, and maxillary palps length has not been either analysed in the literature about cave beetles, but investigations on the diet of these species would bring interesting insight into the adaptive nature of maxillary palps elongation.

Second, our analyses of the syntopic sister species pair *A*. *harbichi* & *A*. *weiratheri* suggests that these two species have undergone strong divergent evolution in pronotum shape (2.6 times greater than in the rest of the genus; P = 0.014), with *A*. *harbichi*, which has a more strongly constricted pronotum, showing the greatest disparity in phylomorphospace (Fig 7). This suggests strong character divergence during or after the speciation process. Character divergence between two closely related species in syntopy could be caused either by (i) reproductive character displacement (if it takes place as part of the speciation process, it would be termed reinforcement; Butlin, 1987), or (ii) ecological character displacement. Since relative maxillary palps length in these two species is more different than expected (Fig 6A), it could imply possibly different food uptake (in cave Cholevinae, maxillary palp length and structural features are considered indicative of diet; [74]). Pronotum shape, on the other hand, is unlikely to play a role in food or microhabitat specialization but might conceivably play a role during copulation: in Cholevinae, copulation often (but not always, see Juberthie-Jupeau, 1988) involves the male holding the female’s pronotum with his protarsi (Schilthuizen, unpublished observations). For these reasons, we would tentatively suggest that both ecological and reproductive character displacement has occurred in this case.

Third, with regard to troglomorphy, *A*. *hoermanni* evolved distinctly increased leg and antenna length relative to the body size, compared with the other members of *Anthroherpon* (Fig 6). It is difficult to speculate on the causes for this, since the exact selection pressures driving the elongation of appendages in troglobites is not fully understood. It might be that the caves where *A*. *hoermanni* live are particularly poor in nutrients, which require the beetles to move faster and travel longer distances, selecting for longer legs and antennae (the former for increased locomotion, the latter for surface increase of sensillae for long-distance detection of food).

*Anthroherpon* is an entirely troglobitic genus, and it is most parsimonious to presume that its ancestor also was a troglobite, since the genus is embedded in a larger clade consisting of entirely troglobitic genera (*Hadesia*, *Leptomeson*, and *Graciliella*). This implies that, in spite of their presumed low mobility and the fragmentary nature of their habitats, troglobitic lineages can, in fact, disperse and diversify over a large geographic area during long periods of time.

In *Anthroherpon*, this certainly is the case. It is the most widely distributed genus of the subtribe Anthroherponina, covering a latitudinal range of more than 200 km and a longitudinal range of more than 170 km. Such “wide” distribution ranges of ancient troglobitic lineages might imply multiple independent colonisations and subsequent extinction of the epigean ancestors [1, 19, 20]. In *Anthroherpon* this is obviously not a possibility because it is embedded in a very large clade consisting of exclusively troglobitic species.

Moreover, until recently, the widely accepted view regarding the radiation of troglobites was that once a lineage has adapted to the subterranean environment within a karst unit, it is unable to expand or diversify over a larger area and, as a result, it remains restricted to a very small range [1, 17, 19]. Many taxa within the genus *Anthroherpon* are short-range endemics: from 26 species and 55 subspecies of *Anthroherpon*, 16 species and 24 subspecies are only known from a single cave. This endemicity, paired with the wide range of the genus as a whole, is a paradox: highly reduced dispersal abilities within the subterranean realm [16, 75] seem to be balanced by occasional long-distance dispersal.

The biogeographic analysis in BioGeoBears shows that models that include founder events (specified by the parameter *j*) have a better fit with the data. Overall, the phylogenetic and biogeographic reconstructions are consistent with the idea that *Anthroherpon* radiated underground from an already troglobitic common ancestor. The founder event signal suggests that speciation was often initiated by long-distance dispersal of one or a few colonists.

Such a scenario of evolutionary radiation accompanied by long-distance founder-events in troglobites has traditionally been interpreted as signifying a peripatric speciation process, involving the genetic revolutions in the small founder populations, followed by stasis when the population size had grown [17, 75]. On the other hand, this need not be the case: (weak) selection in large, isolated populations, may, over the long time periods that our dated phylogeny implies, also cause speciation [76].

The observed phylogeographical patterns in *Anthroherpon* reflect a complex of paleo-biogeographical factors, involving the geological history of the Dinaric Mountains. So far, similar studies of Dinaric terrestrial troglobites are lacking, so we cannot make a comparison with other groups to check for concordance with our results. However, studies on Dinaric stygobites indicate that their distribution patterns do not correspond to recent hydrological divisions but were attained in past drainage areas in geological history and preserved until today [31]. A recent study of *Congeria* Partsch, 1835, the only known troglobitic bivalve, suggests that it separated from its closest relative approximately at the same time as *Anthroherpon* began to diverge (22–23 MYA)[77]. Since there is no reliable dating for vicariant events or the age of subterranean habitats of the Dinaric Mountains that could be used as a calibration point, dating phylogenies of Dinaric troglobites is problematic. Trontelj et al. [45] have estimated the timeframe of the cladogenetic events for the aquatic isopod *Asellus aquaticus* Linnaeus, the cave salamander *Proteus anguinus* Laurenti, and the cave shrimp *Troglocharis* Dormitzer. They also encountered an inability to find reliable time estimates for paleogeographic events to calibrate local molecular clocks for different lineages [45]. In our case, we alleviated this problem by using the separation of the Sardinian microplates from mainland Europe to calibrate the phylogeny of Western Mediterranean Leptodirini [3].

Although our comprehensive study has allowed a more focused evaluation of competing hypotheses about the evolutionary radiation in *Anthroherpon*, additional targeted studies will be needed to confirm some of the conclusions reached here. Further molecular studies may be particularly helpful. For example, niche differentiation in syntopic species pairs could be assessed with metabarcoding of gut contents [78]. Also, studies of genetic polymorphisms in large population samples will allow calculations of historical gene flow and ancestral population sizes for endemic species, enabling an evaluation of the potential role of founder events and bottlenecks [79].

## Supporting information

S1 Table Sequenced specimens, with accession numbers, depository, locality, and collectors. All vouchers are stored at CINJ.
(XLSX)

S2 Table Distances between areas in km. Abbreviations of geographic areas as in Figs 3 and 4.
(TIF)

S3 Table The list of material included in the morphometric analyses. Abbreviations: CINJ (collection Iva Njunjić), CMPR (collection Michel Perreau), CDP (collection Dragan Pavićević), MNHN (collection Muséum national d’histoire naturelle), CG (Crna Gora), BIH (Bosnia and Herzegovina), CRO (Croatia).
(DOCX)

S4 Table The list of morphometric data. Abbreviations: palp2-4: length of maxillary palps 2–4 (1^st^ maxillary palp was not visible in most mounted specimens so we didn’t measure its length); Ant 1–11: length of antennomerae 1–11; L1tib: length of protibia; L2tib: length of mesotibia; L3tib: length of metatibia; L1tarsus1-5: length of 1–5 protarsomere; L2tarsus1-5: length of 1–5 mesotarsomere; L3tarsus1-5: length of 1–5 metatarsomere; Head.lgth: head length; Head.antW: anterior width of the head; Head.postW: posterior width of the head; Pntm.Lgth: length of pronotum; Pntm.antW: anterior width of pronotum; Pntm.postW: posterior width of pronotum; Elyt.Lgth: length of elytra; Meso.Lgth: length of mesothoracic pedunculus.
(XLSX)

S1 Fig Landmarks recorded on the body of *Anthroherpon*. (TIF)

S2 Fig Phylogenetic analysis was performed in BEAST2 for mtDNA and nDNA separately. A. mtDNA, B. nDNA.
(TIF)

S3 Fig Maximum likelihood analysis. (PDF)
