# Supplementary figures and images for: Comprehensive evolutionary analysis of the Anthroherpon radiation (Coleoptera, Leiodidae, Leptodirini)
Source: PLoS One. 2018 Jun 8;13(6):e0198367. doi: 10.1371/journal.pone.0198367 (PMC5993249; doi:10.1371/journal.pone.0198367)

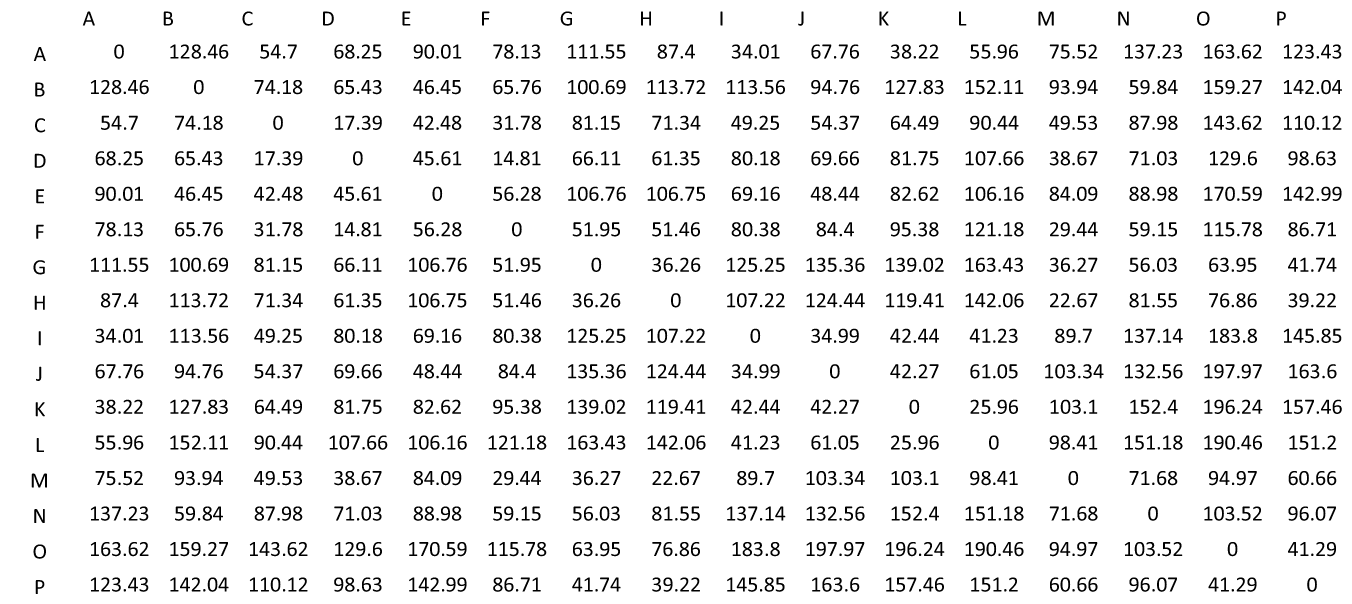

Supplement: S2 Table — Abbreviations of geographic areas as in Figs 3 and 4. (TIF) [file pone.0198367.s002.tif]

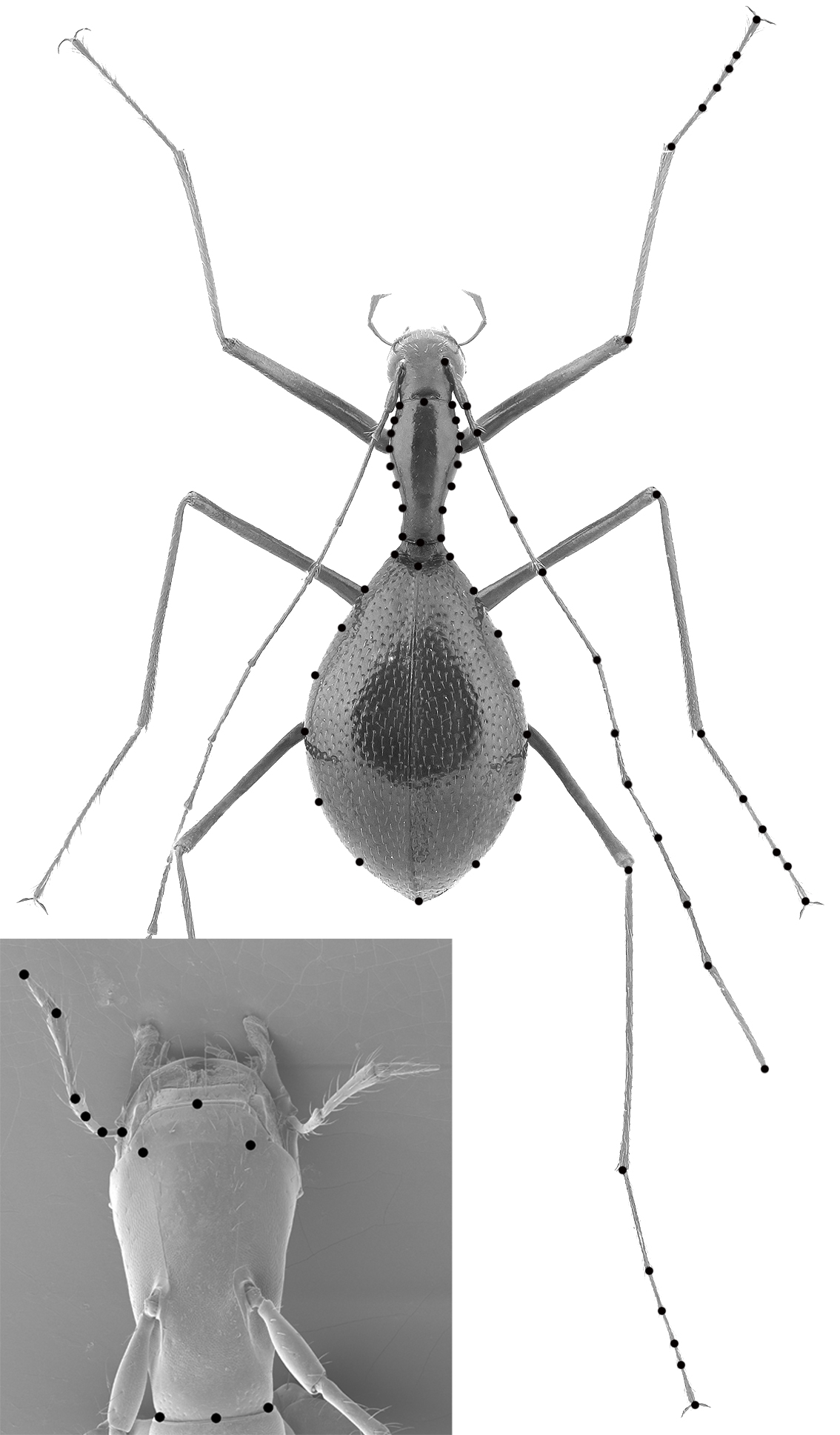

Supplement: S1 Fig — (TIF) [file pone.0198367.s005.tif]

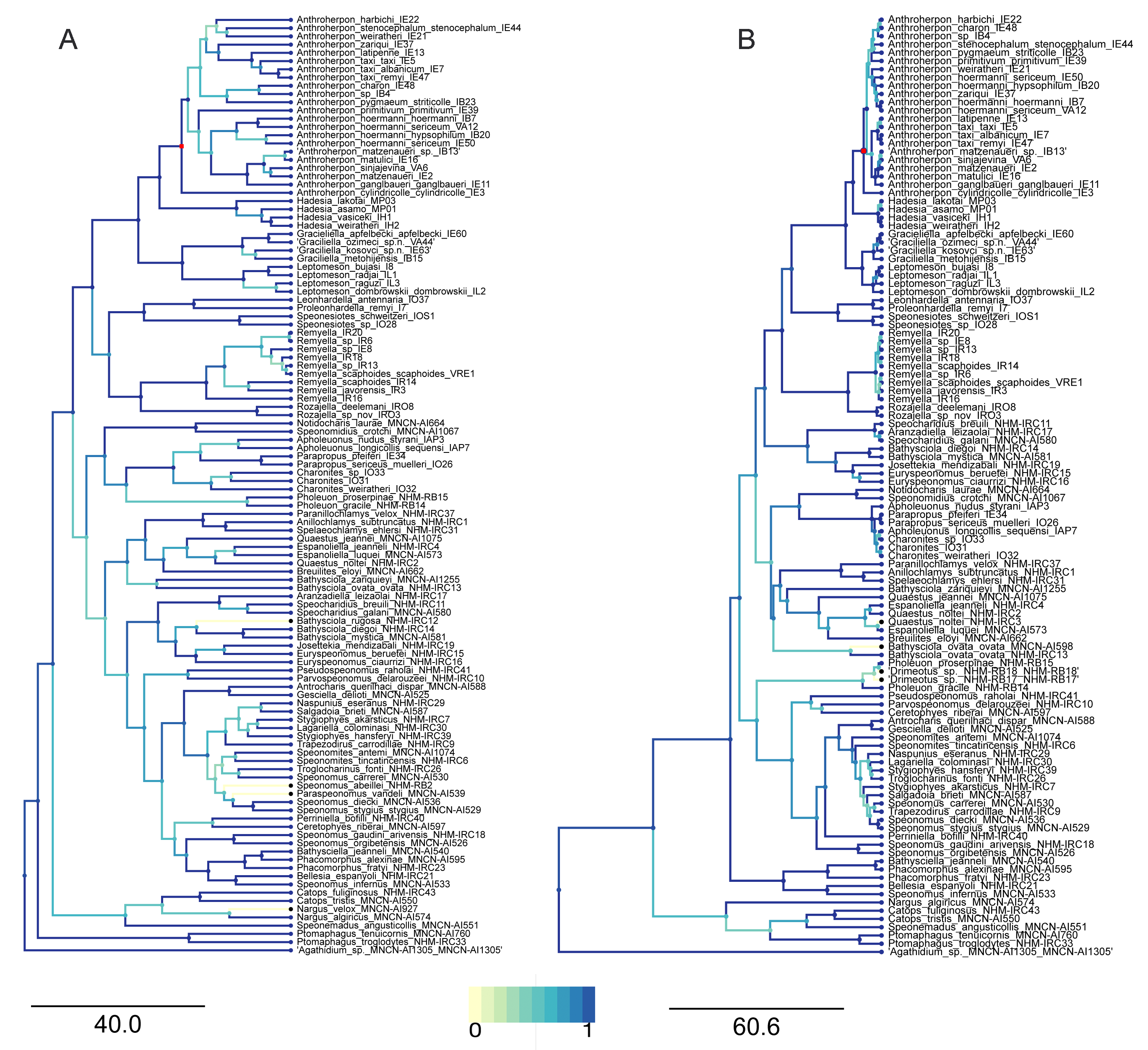

Supplement: S2 Fig — A. mtDNA, B. nDNA. (TIF) [file pone.0198367.s006.tif]

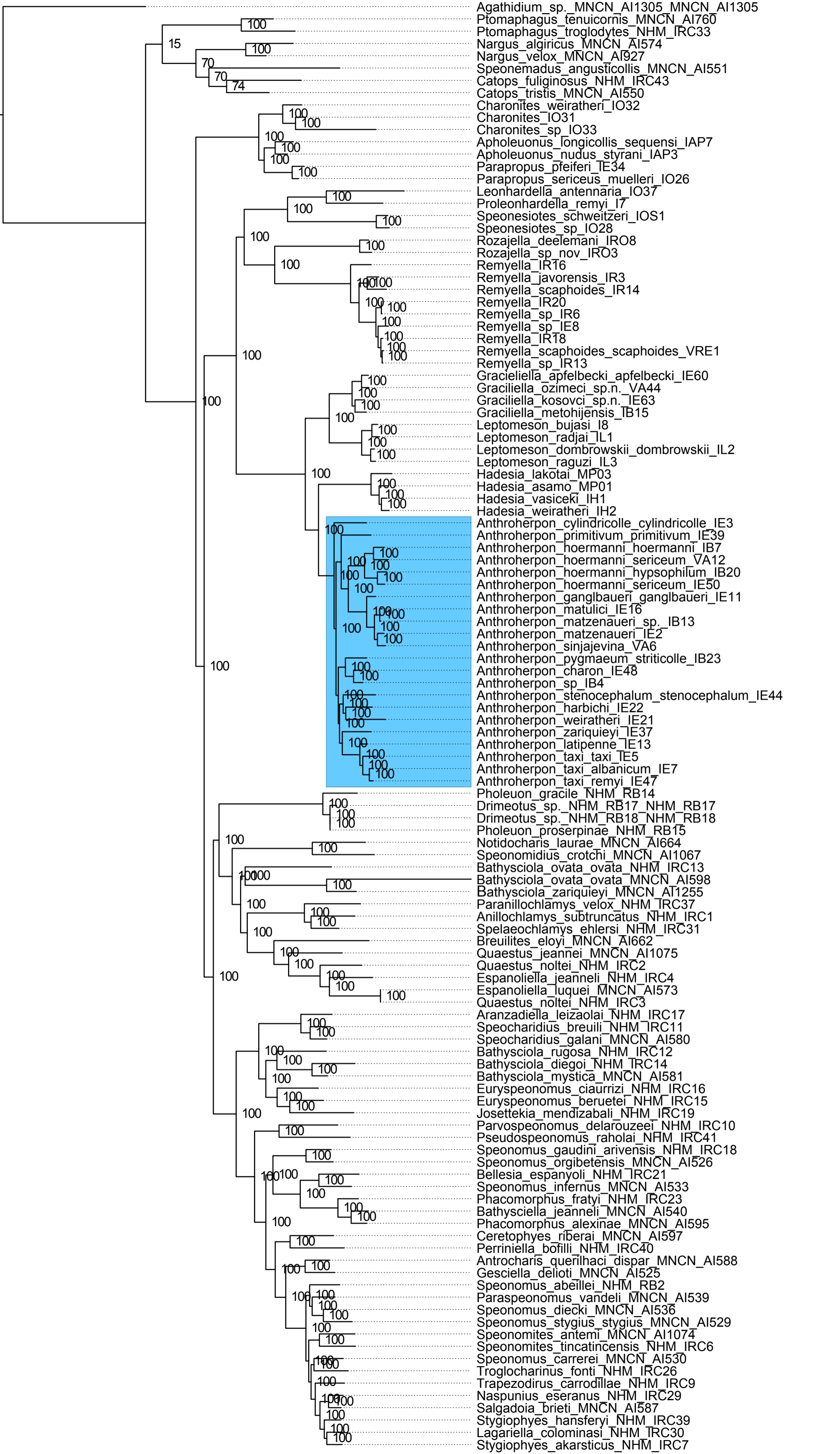

Supplement: S3 Fig — (PDF) [file pone.0198367.s007.pdf]
