# Supplementary material for: Comprehensive evolutionary analysis of the Anthroherpon radiation (Coleoptera, Leiodidae, Leptodirini)
Source: PLoS One. 2018 Jun 8;13(6):e0198367. doi: 10.1371/journal.pone.0198367 (PMC5993249; doi:10.1371/journal.pone.0198367)
Supplement: S3 Table — Abbreviations: CINJ (collection Iva Njunjić), CMPR (collection Michel Perreau), CDP (collection Dragan Pavićević), MNHN (collection Muséum national d’histoire naturelle), CG (Crna Gora), BIH (Bosnia and Herzegovina), CRO (Croatia). (DOCX) [file pone.0198367.s003.docx]

| **Col. Number.** | **Genus** | **Species** | **Subspecies** | **Locality and legator (where available)** |
| --- | --- | --- | --- | --- |
| INJ1 (CINJ) | *Anthroherpon* | *stenocephalum* | *stenocephalum* | BIH, Olovo, Bijambare, I. Njunjić |
| INJ2 (CINJ) | *Anthroherpon* | *taxi* | *remyi* | CG, Berane, Lubnice, Županska pećina, 23.03.2014, I. Njunjić |
| INJ3 (CINJ) | *Anthroherpon* | *taxi* | *remyi* | CG, Berane, Lubnice, Županska pećina, 23.03.2014, I. Njunjić |
| INJ4 (CINJ) | *Anthroherpon* | *taxi* | *remyi* | CG, Berane, Lubnice, Županska pećina, 23.03.2014, I. Njunjić |
| INJ5 (CINJ) | *Anthroherpon* | *taxi* | *remyi* | CG, Berane, Lubnice, Županska pećina, 23.03.2014, I. Njunjić |
| INJ6 (CINJ) | *Anthroherpon* | *taxi* | *remyi* | CG, Berane, Lubnice, Županska pećina, 23.03.2014, I. Njunjić |
| INJ7 (CINJ) | *Anthroherpon* | *cylindricolle* | *cylindricolle* | BIH, Rogatica, Golubovići, Golubovića pećina, 02.05.2013, P. Kosovac |
| INJ8 (CINJ) | *Anthroherpon* | *cylindricolle* | *cylindricolle* | BIH, Rogatica, Golubovići, Golubovića pećina, 02.05.2013, P. Kosovac |
| INJ9 (CINJ) | *Anthroherpon* | *cylindricolle* | *cylindricolle* | BIH, Rogatica, Golubovići, Golubovića pećina, 02.05.2013, P. Kosovac |
| INJ10 (CINJ) | *Anthroherpon* | *cylindricolle* | *cylindricolle* | BIH, Rogatica, Golubovići, Golubovića pećina, 02.05.2013, P. Kosovac |
| INJ11 (CINJ) | *Anthroherpon* | *cylindricolle* | *cylindricolle* | BIH, Rogatica, Golubovići, Golubovića pećina, 02.05.2013, P. Kosovac |
| INJ12 (CINJ) | *Anthroherpon* | *stenocephalum* | *stenocephalum* | BIH, Olovo, Bijambare, 04.05.2013, I. Njunjić |
| INJ13 (CINJ) | *Anthroherpon* | *stenocephalum* | *stenocephalum* | BIH, Olovo, Bijambare, 04.05.2013, I. Njunjić |
| INJ14 (CINJ) | *Anthroherpon* | *stenocephalum* | *stenocephalum* | BIH, Olovo, Bijambare, 04.05.2013, I. Njunjić |
| INJ15 (CINJ) | *Anthroherpon* | *stenocephalum* | *stenocephalum* | BIH, Olovo, Bijambare, 04.05.2013, I. Njunjić |
| INJ16 (CINJ) | *Anthroherpon* | *cylindricolle* | *thoracicum* | BIH, Pale, Careve vode, Novakova pećina, 30.08.2013. |
| INJ17 (CINJ) | *Anthroherpon* | *cylindricolle* | *thoracicum* | BIH, Pale, Careve vode, Novakova pećina, 30.08.2013. |
| INJ18 (CINJ) | *Anthroherpon* | *cylindricolle* | *thoracicum* | BIH, Pale, Careve vode, Novakova pećina, 30.08.2013. |
| INJ19 (CINJ) | *Anthroherpon* | *cylindricolle* | *thoracicum* | BIH, Pale, Careve vode, Novakova pećina, 30.08.2013. |
| INJ20 (CINJ) | *Anthroherpon* | *cylindricolle* | *thoracicum* | BIH, Pale, Careve vode, Novakova pećina, 30.08.2013. |
| INJ21 (CINJ) | *Anthroherpon* | *sinjajevina* |  | CG, Sinjajevina, Rudanca, Blažova pećina, 24.06.2013. I. Njunjić |
| INJ22 (CINJ) | *Anthroherpon* | *sinjajevina* |  | CG, Sinjajevina, Rudanca, Blažova pećina, 24.06.2013, I. Njunjić |
| INJ23 (CINJ) | *Anthroherpon* | *sinjajevina* |  | CG, Sinjajevina, Rudanca, Blažova pećina, 24.06.2013, P. Kosovac |
| INJ24 (CINJ) | *Anthroherpon* | *cylindricolle* | *cylindricolle* | BIH, Rogatica, Golubovići, Golubovića pećina, 30.08.2013, P. Kosovac |
| INJ25 (CINJ) | *Anthroherpon* | *cylindricolle* | *cylindricolle* | BIH, Rogatica, Golubovići, Golubovića pećina, 30.08.2013, P. Kosovac |
| INJ26 (CINJ) | *Anthroherpon* | *cylindricolle* | *cylindricolle* | BIH, Rogatica, Golubovići, Golubovića pećina, 30.08.2013, P. Kosovac |
| INJ27 (CINJ) | *Anthroherpon* | *cylindricolle* | *cylindricolle* | BIH, Rogatica, Golubovići, Golubovića pećina, 30.08.2013, P. Kosovac |
| INJ28 (CINJ) | *Anthroherpon* | *hoermanni* | *hoermanni* | BIH, Kalinovik, Borija, Borija pećina, 20.06.2013, I. Njunjić |
| INJ29 (CINJ) | *Anthroherpon* | *hoermanni* | *hoermanni* | BIH, Kalinovik, Borija, Borija pećina, 20.06.2013, I. Njunjić |
| INJ30 (CINJ) | *Anthroherpon* | *hoermanni* | *hoermanni* | BIH, Kalinovik, Borija, Borija pećina, 20.06.2013, I. Njunjić |
| INJ31 (CINJ) | *Anthroherpon* | *charon* |  | BIH, Trebević, Luke, Ledenica,17.06.2013, I. Njunjić |
| INJ32 (CINJ) | *Graciliella* | *metohijensis* |  | Hercegovina, Korita, Kobilja glava, Veliko Đatlo, 13.09.2006, M. Perreau |
| INJ33 (CINJ) | *Graciliella* | *metohijensis* |  | Hercegovina, Nevesinje, Balabani, Drvendžina pećina, 21.12.2003, M. Đokić |
| INJ34 (CINJ) | *Graciliella* | *metohijensis* |  | Hercegovina, Nevesinje, Balabani, Drvendžina pećina, 21.12.2003, M. Đokić |
| INJ35 (CINJ) | *Anthroherpon* | *charon* |  | BIH, Trebević, Luke, Ledenica,17.06.2013, I. Njunjić |
| INJ36 (CINJ) | *Anthroherpon* | *charon* |  | BIH, Trebević, Luke, Ledenica,17.06.2013, I. Njunjić |
| INJ37 (CINJ) | *Anthroherpon* | *charon* |  | BIH, Trebević, Luke, Ledenica,17.06.2013, I. Njunjić |
| INJ38 (CINJ) | *Anthroherpon* | *harbichi* |  | BIH, Sarajevo, Crepoljsko polje, Kečina stena, 01.05-18.06.2013, I. Njunjić |
| INJ39 (CINJ) | *Anthroherpon* | *harbichi* |  | BIH, Sarajevo, Crepoljsko polje, Kečina stena, 01.05-18.06.2013, I. Njunjić |
| INJ40 (CINJ) | *Anthroherpon* | *harbichi* |  | BIH, Sarajevo, Crepoljsko polje, Kečina stena, 05.2013, I. Njunjić |
| INJ41 (CINJ) | *Anthroherpon* | *harbichi* |  | BIH, Sarajevo, Crepoljsko polje, Kečina stena, 05.2013, I. Njunjić |
| INJ42 (CINJ) | *Anthroherpon* | *harbichi* |  | BIH, Sarajevo, Crepoljsko polje, Kečina stena, 01.09.2013, I. Njunjić |
| INJ43 (CINJ) | *Anthroherpon* | *sinjajevina* |  | MNE, Sinjajevina, Rudanca, Blažova pećina, 24.06.2013, I. Njunjić |
| INJ44 (CINJ) | *Anthroherpon* | *sinjajevina* |  | MNE, Sinjajevina, Rudanca, Blažova pećina, 24.06.2013, I. Njunjić |
| INJ45 (CINJ) | *Graciliella* | *ozimeci* |  | CRO, Dubrovnik, Točionik, Vranja pećina, 08.11.2014, R. Ozimec |
| INJ46 (CINJ) | *Graciliella* | *ozimeci* |  | CRO, Dubrovnik, Točionik, Vranja pećina, 08.11.2014, R. Ozimec |
| INJ47 (CINJ) | *Graciliella* | *ozimeci* |  | CRO, Dubrovnik, Točionik, Vranja pećina, 08.11.2014, R. Ozimec |
| INJ48 (CINJ) | *Graciliella* | *ozimeci* |  | CRO, Dubrovnik, Točionik, Vranja pećina, 08.11.2014, R. Ozimec |
| INJ49 (CINJ) | *Graciliella* | *ozimeci* |  | CRO, Dubrovnik, Točionik, Vranja pećina, 08.11.2014, R. Ozimec |
| INJ50 (CINJ) | *Graciliella* | *ozimeci* |  | MNE, Orjen, Grahovo, Vojvode Dakovića, 22.08.2007, M. Perreau |
| INJ51 (CINJ) | *Graciliella* | *ozimeci* |  | CRO, Dubrovnik, Točionik, Vranja pećina, 08.11.2014, R. Ozimec |
| INJ52 (CINJ) | *Graciliella* | *ozimeci* |  | CRO, Dubrovnik, Točionik, Vranja pećina, 08.11.2014, R. Ozimec |
| INJ53 (CINJ) | *Graciliella* | *ozimeci* |  | CRO, Dubrovnik, Točionik, Vranja pećina, 08.11.2014, R. Ozimec |
| INJ54 (CINJ) | *Graciliella* | *kosovaci* |  | CRO, Tunel Konavle, Kaverna 781, 09.11.2014, R. Ozimec |
| INJ55 (CINJ) | *Gracileilla* | *metohijensis* |  | Hercegovina, Nevesinje, Balabani, Drvendžina pećina, 26.08.2013, I. Njunjić |
| INJ56 (CINJ) | *Graciliella* | *metohijensis* |  | Hercegovina, Nevesinje, Balabani, Drvendžina pećina, 26.08.2013, I. Njunjić |
| INJ57 (CINJ) | *Graciliella* | *metohijensis* |  | Hercegovina, Nevesinje, Balabani, Drvendžina pećina, 26.08.2013, I. Njunjić |
| INJ58 (CINJ) | *Graciliella* | *apfelbecki* |  | CRO, Bobani, Grabovica, 08.05.2011, R. Ozimec |
| INJ59 (CINJ) | *Graciliella* | *metohijensis* |  | Hercegovina, Nevesinje, Balabani, Drvendžina pećina, 26.08.2013, I. Njunjić |
| INJ60 (CINJ) | *Graciliella* | *metohijensis* |  | Hercegovina, Nevesinje, Balabani, Drvendžina pećina, 26.08.2013, I. Njunjić |
| INJ61 (CINJ) | *Graciliella* | *metohijensis* |  | Hercegovina, Nevesinje, Balabani, Drvendžina pećina, 26.08.2013, I. Njunjić |
| INJ62 (CINJ) | *Graciliella* | *metohijensis* |  | Hercegovina, Nevesinje, Balabani, Drvendžina pećina, 26.08.2013, I. Njunjić |
| INJ63 (CINJ) | *Graciliella* | *metohijensis* |  | Hercegovina, Nevesinje, Balabani, Drvendžina pećina, 26.08.2013, I. Njunjić |
| INJ64 (CINJ) | *Graciliella* | *metohijensis* |  | Hercegovina, Nevesinje, Balabani, Drvendžina pećina, 26.08.2013, I. Njunjić |
| INJ65 (CINJ) | *Graciliella* | *metohijensis* |  | Hercegovina, Nevesinje, Balabani, Drvendžina pećina, 26.08.2013, I. Njunjić |
| INJ66 (CINJ) | *Graciliella* | *metohijensis* |  | Hercegovina, Nevesinje, Balabani, Drvendžina pećina, 26.08.2013, I. Njunjić |
| INJ67 (CINJ) | *Graciliella* | *metohijensis* |  | Hercegovina, Nevesinje, Balabani, Drvendžina pećina, 26.08.2013, I. Njunjić |
| INJ68 (CINJ) | *Graciliella* | *metohijensis* |  | Hercegovina, Nevesinje, Balabani, Drvendžina pećina, 26.08.2013, I. Njunjić |
| INJ69 (CINJ) | *Graciliella* | *ozimeci* |  | CRO, Dubrovnik, Točionik, Vranja pećina, 08.11.2014, R. Ozimec |
| INJ70 (CINJ) | *Graciliella* | *ozimeci* |  | CRO, Dubrovnik, Točionik, Vranja pećina, 08.11.2014, R. Ozimec |
| INJ71 (CINJ) | *Graciliella* | *apfelbecki* | *apfelbecki* | CRO, Gromača, Špilja za Gromačkom vlakom, 28.08.2014, R. Ozimec |
| INJ72 (CINJ) | *Anthroherpon* | *primitivum* |  | BIH, Trebinje, Turica, Mravinjac, 31.07.2013, M. Perreau |
| INJ73 (CINJ) | *Anthroherpon* | *primitivum* |  | BIH, Trebinje, Turica, Mravinjac, 31.07.2013, M. Perreau |
| INJ74 (CINJ) | *Anthroherpon* | *primitivum* |  | BIH, Trebinje, Turica, Mravinjac, 31.07.2013, M. Perreau |
| INJ75 (CINJ) | *Anthroherpon* | *primitivum* |  | BIH, Trebinje, Turica, Mravinjac, 31.07.2013, M. Perreau |
| INJ76 (CINJ) | *Anthroherpon* | *taxi* | *albanicum* | Prokletije, Ćaf Borit, Kolektor, 24.07.2012, M. Perreau |
| INJ77 (CINJ) | *Anthroherpon* | *taxi* | *albanicum* | Prokletije, Ćaf Borit, Kolektor, 24.07.2012, M. Perreau |
| INJ78 (CINJ) | *Anthroherpon* | *taxi* | *albanicum* | Prokletije, Ćaf Borit, Kolektor, 24.07.2012, M. Perreau |
| INJ79 (CINJ) | *Anthroherpon* | *zariquieyi* |  | MNE, Durmitor, Pećina u Sedlenoj gredi, 15.07.2014, I. Njunjić |
| INJ80 (CINJ) | *Anthroherpon* | *zariquieyi* |  | MNE, Durmitor, Pećina u Sedlenoj gredi, 15.07.2014, I. Njunjić |
| INJ81 (CINJ) | *Anthroherpon* | *zariquieyi* |  | MNE, Durmitor, Pećina u Sedlenoj gredi, 15.07.2014, I. Njunjić |
| INJ82 (CINJ) | *Anthroherpon* | *hoermanni* | *hypsophilum* | Hercegovina, Lebršnik, Dvogrla jama, 18.09.2006, M. Perreau |
| INJ83 (CINJ) | *Anthroherpon* | *hoermanni* | *hypsophilum* | Hercegovina, Lebršnik, Dvogrla jama, 18.09.2006, M. Perreau |
| INJ84 (CINJ) | *Anthroherpon* | *hoermanni* | *hypsophilum* | Hercegovina, Lebršnik, Vilina pećina,09.09.2006. M. Perreau |
| INJ85 (CINJ) | *Anthroherpon* | *hoermanni* | *hypsophilum* | Hercegovina, Lebršnik, Dvogrla jama, 18.09.2006, M. Perreau |
| INJ86 (CINJ) | *Anthroherpon* | *hoermanni* | *hypsophilum* | Hercegovina, Lebršnik, Dvogrla jama, 18.09.2006, M. Perreau |
| INJ87 (CINJ) | *Anthroherpon* | *hoermanni* | *hypsophilum* | Hercegovina, Lebršnik, Čavčarica jama, 09.09.2005, M. Perreau |
| INJ88 (CINJ) | *Anthroherpon* | *hoermanni* | *hoermanni* | BIH, Zelengora, Nedavići, Vareničina pećina, 21.06.2013, I. Njunjić |
| INJ89 (CINJ) | *Graciliella* | *metohijensis* |  | CG, Somina, Donje Čarađe, Prljača, 06.08.2010, S. Ognjenović |
| INJ90 (CINJ) | *Graciliella* | *apfelbecki* |  | Hercegovina, Bileća, Bijele Rudine, jama Vranjača, 07.06.2005, M. Perreau |
| INJ91 (CINJ) | *Graciliella* | *apfelbecki* |  | Hercegovina, Bileća, Bijele Rudine, jama Vranjača, 07.06.2005, M. Perreau |
| INJ92 (CINJ) | *Graciliella* | *ganglbaueri* | *ganglbaueri* | BIH, Nevesinje, Bišina, Novakuša, 21.06.2013, I. Njunjić |
| INJ93 (CINJ) | *Anthroherpon* | *ganglbaueri* | *ganglbaueri* | BIH, Nevesinje, Bišina, Novakuša, 21.06.2013, I. Njunjić |
| INJ94 (CINJ) | *Anthroherpon* | *ganglbaueri* | *ganglbaueri* | BIH, Nevesinje, Bišina, Novakuša, 21.06.2013, I. Njunjić |
| INJ95 (CINJ) | *Graciliella* | *apfelbecki* | *lahneri* | CG, Virpazar, env. Trnovo |
| INJ96 (CINJ) | *Graciliella* | *apfelbecki* | *scutulatum* | Bravenik |
| INJ97 (CINJ) | *Anthroherpon* | *matzenaueri* | *taliensis* | CG, Maganik, Kapetanovo jezero, Dola pećina, 01.08.2013, Mp. Perreau |
| INJ98 (CINJ) | *Anthroherpon* | *cecai* |  | CG, Durmitor, Mala Crna Gora, Jama u Podu, 03.08.1992, A. Milosavljević |
| INJ99 (CINJ) | *Anthroherpon* | *taxi* | *sydowi* | CG, Tali Mt, Mlječikova pećina, 03.08.2013, M. Perreau |
| INJ100 (CINJ) | *Anthroherpon* | *latipenne* |  | CG, Somina, Donje Čarađe, Prljača, 06.08.2010, S. Ognjenović |
| INJ101 (CINJ) | *Anthroherpon* | *latipenne* | *punctipennis* | CG, Maganik, Kapetanovo jezero, Dola pećina, 01.08.2013, Mp. Perreau |
| INJ102 (CINJ) | *Anthroherpon* | *taxi* | *albanicum* | CG, Prokletije, ćaf Borit, Jama kolektor, 25.07.2011-24.07.2012, M. Popović |
| INJ103 (CINJ) | *Anthroherpon* | *taxi* | *albanicum* | CG, Prokletije, ćaf Borit, Jama kolektor, 25.07.2011-24.07.2012, M. Popović |
| INJ104 (CINJ) | *Anthroherpon* | *taxi* | *taxi* | CG, Orjen, Kameno more, Dvestotka, 13.08.2009, I. Njunjić |
| INJ105 (CINJ) | *Graciliella* | *apfelbecki* | *lahneri* | CG, Trnovo, Grbočica, 28.04.2004. |
| INJ106 (CINJ) | *Anthroherpon* | *ganglbaueri* | *ganglbaueri* | BIH, Nevesinje, Bišina, Novakuša, 21.06.2013, I. Njunjić |
| INJ107 (CINJ) | *Anthroherpon* | *ganglbaueri* | *ganglbaueri* | BIH, Nevesinje, Bišina, Novakuša, 21.06.2013, I. Njunjić |
| INJ108 (CINJ) | *Anthroherpon* | *ganglbaueri* | *ganglbaueri* | BIH, Nevesinje, Bišina, Novakuša, 21.06.2013, I. Njunjić |
| INJ109 (CINJ) | *Anthroherpon* | *ganglbaueri* | *ganglbaueri* | BIH, Nevesinje, Bišina, Novakuša, 21.06.2013, I. Njunjić |
| INJ110 (CINJ) | *Anthroherpon* | *latipenne* |  | CG, Orjen, Križeva jama |
| INJ111 (CINJ) | *Anthroherpon* | *latipenne* |  | CG, Orjen, Križeva jama |
| INJ112 (CINJ) | *Anthroherpon* | *latipenne* |  | CG, Somina, Donje Čarađe, Prljača, 06.08.2010, S. Ognjenović |
| INJ113 (CINJ) | *Anthroherpon* | *latipenne* |  | CG, Somina, Donje Čarađe, Prljača, 06.08.2010, S. Ognjenović |
| INJ114 (CINJ) | *Anthroherpon* | *taxi* | *taxi* | CG, Orjen, Kameno more, Dvestotka, 13.08.2009, I. Njunjić |
| INJ115 (CINJ) | *Anthroherpon* | *taxi* | *taxi* | CG, Orjen, Kameno more, Dvestotka, 13.08.2009, I. Njunjić |
| INJ116 (CINJ) | *Anthroherpon* | *pygmaeum* | *stricticolle* | BIH, Bjelašnica, Sudareva pećina, 03.09.2013, I. Njunjić |
| INJ117 (CINJ) | *Anthroherpon* | *pygmaeum* | *stricticolle* | BIH, Bjelašnica, Sudareva pećina, 03.09.2013, I. Njunjić |
| INJ118 (CINJ) | *Anthroherpon* | *pygmaeum* | *stricticolle* | BIH, Bjelašnica, Sudareva pećina, 03.09.2013, I. Njunjić |
| INJ119 (CINJ) | *Anthroherpon* | *pygmaeum* | *stricticolle* | BIH, Bjelašnica, Sudareva pećina, 03.09.2013, I. Njunjić |
| INJ120 (CINJ) | *Anthroherpon* | *pygmaeum* | *stricticolle* | BIH, Bjelašnica, Sudareva pećina, 03.09.2013, I. Njunjić |
| INJ121 (CINJ) | *Anthroherpon* | *taxi* | *taxi* | CG, Orjen, Dvestotka, 13.08.2009, I. Njunjić |
| INJ122 (CINJ) | *Anthroherpon* | *taxi* | *taxi* | CG, Orjen, Dvestotka, 13.08.2009, I. Njunjić |
| INJ123 (CINJ) | *Anthroherpon* | *hoermanni* | *hoermanni* | BIH, Kalinovik, Borija, Borija pećina, 20.06.2013, I. Njunjić |
| INJ124 (CINJ) | *Anthroherpon* | *taxi* | *taxi* | CG, Orjen, Dvestotka, 13.08.2009, I. Njunjić |
| INJ125 (CINJ) | *Anthroherpon* | *taxi* | *taxi* | CG, Orjen, Dvestotka, 13.08.2009, I. Njunjić |
| INJ126 (CINJ) | *Anthroherpon* | *taxi* | *albanicum* | Prokletije, Ćaf Borit, Babina sisa, 15.07.2012, M. Perreau |
| INJ127 (CINJ) | *Anthroherpon* | *taxi* | *albanicum* | Prokletije, Ćaf Borit, Babina sisa, 15.07.2012, M. Perreau |
| INJ128 (CINJ) | *Graciliella* | *metohijensis* |  | BIH, Hercegovina, Korita, Kobilja glava, Đatlo, 05.08.2013, M. Perreau |
| INJ129 (CINJ) | *Anthroherpon* | *zariquieyi* |  | CG, Durmitor, Sedlo, Pećina u Sedlenoj gredi, 24.06.2013, I.Njunjić |
| IE44 (CINJ) | *Anthroherpon* | *stenocephalum* | stenocephalum | BIH, Olovo, Bijambare, 04.05.2013, I. Njunjić |
| VA5 (CINJ) | *Anthroherpon* | *erebus* | scheibeli | BIH, Trnovo, Bjeličina pećina |
| MP1 (CMPR) | *Graciliella* | *apfelbecki* |  | BIH, Popovo polje, Vjeternica, M. Perreau |
| MP2 (CMPR) | *Graciliella* | *apfelbecki* |  | BIH, Popovo polje, Vjeternica, M. Perreau |
| MP3 (CMPR) | *Anthroherpon* | *latipenne* | *latipenne* | Hercegovina, Gubar Mt, Bukova rupa, 11.09.2008. M. Perreau |
| MP4 (CMPR) | *Anthroherpon* | *latipenne* | *latipenne* | Hercegovina, Gubar Mt, Bukova rupa, 11.09.2008. M. Perreau |
| MP5 (CMPR) | *Graciliella* | *metohijensis* |  | BIH, Brestica near Korita, Jametina jama, 14.08.2007, M. Perreau |
| MP6 (CMPR) | *Graciliella* | *metohijensis* |  | BIH, Brestica near Korita, Jametina jama, 14.08.2007, M. Perreau |
| MNHN | *Anthroherpon* | *charon* |  | Trebević, Reitter |
| MNHN | *Anthroherpon* | *charon* |  | Trebević, Reitter |
| MNHN | *Anthroherpon* | *charon* |  | Ledenjača, Trebević, Svirčev 11.29. |
| MNHN | *Anthroherpon* | *erebus* | *scheibeli* | Trnovo (type) |
| MNHN | *Anthroherpon* | *erebus* | *scheibeli* | Trnovo (cotype) |
| MNHN | *Anthroherpon* | *erebus* | *scheibeli* | Trnovo |
| MNHN | *Anthroherpon* | *primitivum* |  | Heregovina, Nevada, Grebci, Čelina pećina, 16.07.1930, Svirčev |
| MNHN | *Anthroherpon* | *weiratheri* |  | BIH, Sarajevo, Kečina stena |
| MNHN | *Anthroherpon* | *weiratheri* |  | BIH, Sarajevo, Kečina stena |
| MNHN | *Graciliella* | *apfelbecki* |  | BIH, Zavala, Vjeternica (cotype) |
| MNHN | *Graciliella* | *apfelbecki* |  | Popovo polje, Grabovica |
| MNHN | *Graciliella* | *apfelbecki* |  | Dubrovnik, Močiljska pećina |
| MNHN | *Graciliella* | *apfelbecki* |  | Dubrovnik, Močiljska pećina |
| MNHN | *Graciliella* | *apfelbecki* |  | Dubrovnik, Močiljska pećina |
| MNHN | *Graciliella* | *apfelbecki* |  | Hercegovina, Grebci, Kali pećina |
